# Micro-Organisms of the Mouth

**Published:** 1907-12

**Authors:** C. L. Ameth

**Affiliations:** Pensacola, Fla.


### MICRO-ORGANISMS OF THE MOUTH.

By Dr. C. L. Ameth, Pensacola, Fla.

It has been stated that there are more bacteria in the saliva than in an equal amount of sewerage. At first thought this statement seems greatly exaggerated but its thruthfulness can be easily demonstrated by anyone who will place a small bit of saliva under the microscope and see the thousands of bacteria which infest every field. The epithelial cells are seen to be literally covered with these small organisms.

A study of these micro-organisms is not only of great interest but it is of extreme importance to the dentist. In a general way all bacteria are divided into two classes; 1st, those which live on dead organic matter, and 2nd, those which exist on living tissue, and this latter class is therefore spoken of as parasitic or pathogenic bacteria.

Ordinarily the bacteria which live upon dead organic matter are of little interest in the study of diseased conditions of the body, but the bacteria found in the oral cavity present an exception to this rule. In the study of diseased conditions of the mouth it is necessary for the dentist to consider these saprophytic bacteria as well as the pathogenic varieties.

A consideration of the conditions found in the mouth show everything favorable to the growth of bacteria, that is, the mouth serves as an ideal incubator. It presents a warm even temperature, plenty of moisture and nourishment, and a favorable reaction.

It is of interest to the dentist to know that the first discoveries in bacteriology were associated with the oral cavity. In 1683, Antonia Van Leeuwenhoek, of Holland, to whom is credited the discovery of bacteria, reported before the Royal Society of London that in the tartar scraped from the teeth he had discovered special forms of micro-organisms. Since his time and especially during the latter part of the last century these micro-organisms which he observed have been carefully examined and studied by quite a number of investigators and for the study of them from a dental standpoint we are indebted to Miller and Goadby.

Some attempts have been made to classify the different varieties of bacteria which are found in the mouth but it is a task which appears to be impossible to accomplish. The immensity of the task can be appreciated when we remember that at different times it is possible for practically all of the unclassified thousands of varieties of bacteria which exist in the soil, water and air, to appear in the mouth, being taken in with the food or water or inhaled with the air.

The bacteria which grow upon dead organic matter are of importance to the dentist when found in the mouth because of the fact that by growing upon particles of food which have found lodgement about the teeth they produce reactions which are destructive to the tissues. One of the most destructive effects follows the production of acids, principally lactic acid, and these acids by their solvent action first upon the enamel of the teeth, and second upon the dentin produce a rapid destruction of the inorganic structures of the teeth. A solution of the organic structures then follows through the action of the enzymes of other bacteria.

When we come to study these two types of bacteria we find it very difficult to draw a line of demarcation separating pathogenic bacteria from the non-pathogenic(or saprophytic). After the penetration of the enamel is accomplished we all know from experience that the destruction of the dentin follows very rapidly and a microscopical examination shows that this destruction is due to the fact that the bacteria have gained access to the dentinal tubules and hence there is little to impede their rapid progress toward the pulp. It is here that the symbiotic relations of the bacteria are most evident. The bacteria which grow upon the surface make favorable conditions for the growth of bacteria which cannot exist in the presence of air.

We can readily see then how it is possible for the pathogenic and non-pathogenic bacteria to be so intimately associated in this destruction of the enamel and dentine that it is practically impossible to separate one from the other. Many of the bacteria which produce this destruction of dentine have been classified by Goadby under two heads—first, those which are found in the deeper layers of carious dentine, and 2nd, those which are found in the superficial layers. His classification is as follows:

### ACID FORMING BACTERIA.

| | |
|---|---|
| Streptococcus brevis<br>Bacillus necro dentalis<br>Staphylococcus albus | Deep layers of carious dentine. |
| Streptococcus brevis<br>Sacina lutea<br>Sarcina aurantiaca<br>Sarcina alba(Eisenberg)<br>Staphylococcus albus<br>Staphylococcus aurens | Superficial layers of carious dentine. |

### BACTERIA WHICH LIQUIFY DENTINE (decalcified).

| | |
|---|---|
| None isolated as yet. | Deep layers of carious dentine. |
| Bacillus mesentericus ruber<br>Bacillus mesentericus vulgatus<br>Bacillus mesentericus fuscus<br>Bacillus furvus<br>Bacillus gingivae pyogenes<br>Bacillus liquifaciens fluorescens motilis<br>Bacillus subtilis<br>Proteus Zenkeri<br>Bacillus plexiformis | Superficial layers of carious dentine. |

In addition to the above organisms other investigators have described other bacteria as causitive factors in the destruction of dentine. No doubt many others will be reported from time to time by various investigators.

The investigations to show the bacteria found in diseased pulp have not been so successful as the above. It has been demonstrated that the pulp may be diseased and yet no bacteria be present; inflammation and destruction of the pulp tissue being due to the toxines produced by the bacteria located in the carious dentine.

While a number of forms of bacteria have been discovered in the pulp, only a few have been classified and there is much work along this line yet to be done by the scientific dentist. As is well known, the ordinary pus cocci are usually found in acute abscesses but Goadby reports that in alveolar abscesses these organisms are not commonly found. From the severity of many of these abscesses we would naturally conclude that the staphylococcus aureus would be the infective agent yet he reports that in his experience it is the least often met with. In this connection it is also interesting to note that the bacillus coli which is commonly classified as being mildly pathogenic is reported to be present in some severe cases. Its effect in these cases is no doubt due in a great part to its gas producing properties. The yeasts are also found in these conditions but they have not as yet been classified.

Of all the inflammatory conditions of the mouth pyorrhoea alveolaris offers the most difficult problem from a bacteriological standpoint. Examinations of the pus and of the tissues themselves of this disease show quite a variety of microorganisms. No one organism has yet been isolated which has been proven to be the causitive factor in the production of this condition. There seems to be very little doubt but that systemic conditions such as faulty tissue changes are undoubtedly predisposing factors, in fact, there is hardly any doubt but that this is also true of other conditions of the mouth.

These facts impress us with the fact that the successful investigator must not be controlled by one view alone but he must be sure to include every contributing factor toward the production of the diseased condition. And the more we study these conditions, the more we feel the need of a thorough knowledge of the metabolism of the entire body. It is only by knowledge of these tissue changes that we can hope to thoroughly work out the diseased conditions and thus continue the onward progress of our profession toward a higher plane.

A class of bacteria which should be of interest to the dentist and which I am afraid is oftentimes overlooked is that class which is known as the strict pathogenic. These pathogenic bacteria which are often found in the mouth may produce their destructive effect in other portions of the body and because there is no lesion in the oral cavity their presence may not be suspected by the dentist. As an illustration of this, in pulmonary tuberculosis there is rarely any tissue lesion in the oral cavity and yet the saliva of the patient is literally swarming with these disease germs. Therefore for the dentist to be able to recognize such a condition or even suspect the presence of this disease he must familiarize himself with the general symptoms of the disease so that he may be always on his guard to be sure that he does not act as a carrier of this disease to others. The same precautions apply to diphtheria but here in the majority of instances the lesion in the oral cavity will serve as a warning.

With the advance which was made during the latter part of the last century in the discovery of the specific organisms of various diseases many unsuccessful attempts were made to isolate the infective agent of syphilis and from time to time various bacteria were described yet it remained for the renowned and lamented Schaudin to demonstrate that the Spirochaete pallida was in all probability the specific organism of this disease and this view has been confirmed by the experiments of Metschnikoff and others. Like tuberculosis this is a disease with which the dentist should have a thorough knowledge so that he may be at all times on the watch for its lesions in the oral cavity.

In this short paper I have attempted a brief consideration of some of the organisms found in the oral cavity and some of the results produced by them. After all our studies of these organisms we are confronted with the fact that susceptibility and immunity play a great part in the diseased conditions which we find in the mouth. For example it is well known that many animals are found to have food particles lodged about their teeth and yet they exibit no signs of dental caries. Many theories have been advanced and many experiments carried on to account for these differences between human beings and other animals and also between human beings themselves yet the matter is far from being settled.

It therefore behooves each one of us as dentists to add his mite of investigation toward the upbuilding of our profession.

BIBLIOGRAPHY.

Principles of Bacteriology, Abbott.
Manual of Bacteriology, Sternberg.
Pathogenic Bacteria, McFarland.
Mycology of the Mouth, Goadby.

